# Direct Oral Anticoagulants Compared to Warfarin in Patients with Intermediate‐ to High‐Risk Pulmonary Embolism: A Systematic Review and Meta‐Analysis

**DOI:** 10.1002/jha2.70031

**Published:** 2025-06-06

**Authors:** Mohamed Nabil Elshafei, Muhammad Salem, Mutasem Almistarihi, Haider Alabd, Ahmed Khalil, Mohammed Danjuma

**Affiliations:** ^1^ Clinical Pharmacy Department Hamad Medical Corporation Doha Qatar; ^2^ Internal Medicine Department Hamad Medical Corporation Doha Qatar; ^3^ Weill Cornell Medicine‐Qatar Doha Qatar

**Keywords:** DOAC, high‐risk, intermediate‐risk, meta‐analysis, pulmonary embolism, warfarin

## Abstract

**Introduction:**

Despite recent advances in clinical therapeutics in patients with venous thromboembolism, uncertainty remains regarding the optimal anticoagulation strategy in patients with intermediate‐ to high‐risk pulmonary embolism. We aimed to evaluate the direct oral anticoagulants’ effectiveness and safety compared to warfarin in patients with intermediate‐ and high‐risk pulmonary embolism.

**Methods:**

In this meta‐analysis, we systematically searched databases PubMed, EMBASE, Web of Science, and the Cochrane Library from their inception to April 30, 2024, for eligible studies that satisfy inclusion criteria. We included both randomized clinical trials and observational studies reporting on patients diagnosed with intermediate‐risk or high‐risk pulmonary embolism who were prescribed warfarin or direct oral anticoagulants (dabigatran, rivaroxaban, apixaban, or edoxaban). Two independent reviewers extracted relevant data from included studies based on the Preferred Reporting Items for Systematic Reviews and Meta‐Analyses guideline. Pooled odds ratios were derived from the included studies using the random‐effects model. The primary outcomes were the rate of venous thromboembolism recurrence and all‐cause mortality. Secondary outcomes were major and minor bleeding.

**Results:**

Six studies, five observational and one randomized (*n* = 553 patients, out of which 45.7% received advanced therapy), were included in our meta‐analysis. Direct oral anticoagulants were associated with comparable recurrence and mortality rates versus warfarin (odds ratio [OR] 0.72, 95% confidence interval [CI] 0.15–3.43) and (OR 0.57, 95% CI 0.16–2.01), respectively. There was no significant difference in the safety outcomes (major and minor bleeding events), with a trend towards a decreased risk among direct oral anticoagulant treated cohorts (OR 0.3, 95% CI 0.08–1.1) and (OR 0.64, 95% CI 0.24–1.7), respectively.

**Conclusion:**

In patients with intermediate‐ to high‐risk pulmonary embolism, with or without advanced therapy use, stabilized on direct oral anticoagulants, efficacy and safety outcomes were comparable to those on warfarin, with a trend towards less risk of major and minor bleeding events.

**Trial registration:**

International PROSPERO database number: CRD42024499532

## 1 Introduction

Pulmonary embolism (PE) typically arises from the dislodgment of blood clots, known as emboli, primarily from deep vein thrombosis (DVT) in the legs or pelvis. However, PE can also result from other materials entering the pulmonary circulation, albeit less commonly, which include air, fat, or tumor cells [1].

Venous thromboembolism (VTE), including DVT and PE, ranks as the third most common acute cardiovascular syndrome worldwide, following myocardial infarction and stroke [2]. In epidemiological studies, the annual incidence rates for PE typically range from 39 to 115 cases per 100,000 population [3]. Despite initial uncertainty, the clinicopathological classification of various PE phenotypes has recently become a lot clearer. PE is classified into three categories based on hemodynamic stability and the presence of right ventricular (RV) strain: low‐risk, intermediate‐risk (submassive), and high‐risk (massive) [3, 4]. A PE is considered high‐risk (massive) if the patient is hemodynamically unstable, characterized by a systolic blood pressure (SBP) less than 90 mm Hg, or a drop in SBP of 40 mm Hg or more from baseline, or if hypotension requires the administration of inotropes or vasopressors [3, 4]. Submassive or intermediate‐risk PE is defined as PE without systemic hypotension, with Pulmonary Embolism Severity Index (PESI) class III–V or simplified PESI (sPESI) ≥ 1, and with either RV dysfunction or myocardial necrosis. If both of the latter are available, the PE is intermediate‐high. If one or none is available, the PE is intermediate‐low [3]. The intermediate‐risk subtype accounts for 40%–50% of patients presenting with acute PE [5].

Whereas acute patients identified as low‐risk PE can be safely treated with anticoagulation as outpatients [3, 4, 6], the intermediate‐risk PE population presents with varying degrees of symptom severity and usually requires management with a short hospital stay [7]. High‐risk acute PE is associated with a significant risk of short‐term mortality and requires aggressive treatment [1, 3, 4]. Thrombolysis is the mainstay intervention in high‐risk PE and can be administered either systemically or using a catheter‐directed approach [3, 5, 8]. In some cases, surgical embolectomy may also be considered a treatment option. These interventions aim to reduce the clot burden within the pulmonary vasculature and improve hemodynamic stability. Consequently, this will result in decreasing the risk of mortality [3, 5].

Anticoagulation is the cornerstone of treatment in PE. This is achieved via using various anticoagulant medications, including subcutaneous low‐molecular‐weight heparin (LMWH), fondaparinux, intravenous unfractionated heparin (UFH), vitamin K antagonists (VKAs) (predominantly warfarin), or direct oral anticoagulants (DOACs). The European Society of Cardiology (ESC) Guidelines for the diagnosis and management of acute pulmonary embolism recommend an initial treatment approach with LMWH anticoagulation for the first 2–3 days in patients with intermediate‐ to high‐risk PE, which aims to ensure stability before transitioning to oral anticoagulation [3]. This recommendation arises from the exclusion of high‐risk patients from phase III trials evaluating DOACs for pulmonary embolism, restricting data on DOACs efficacy and safety in this subgroup [3, 8, 9]. Additionally, there is a recognized risk of hemodynamic deterioration within the first 24–48 h in about 5% of patients with submassive PE, which may need thrombolysis intervention [10, 11].

There is an uncertainty regarding the optimal timing for transitioning patients from parenteral to oral anticoagulation therapy. However, pharmacokinetic data suggest that DOACs can achieve an anticoagulant effect as rapidly as traditional parenteral agents. Phase III clinical trials have shown the non‐inferior efficacy of using higher doses of apixaban for 7 days or rivaroxaban for 3 weeks in adopting a single oral anticoagulant strategy [9, 12]. Additionally, a retrospective post hoc analysis cohort study was conducted across seven French centers, involving 2411 patients diagnosed with pulmonary embolism (PE) at intermediate risk. It was reported that patients who received DOAC after 72 h of LMWH treatment had a 9% risk of death and shock at 30 days compared with 4.8% in patients who received a DOAC within 72 h of LMWH treatment initiation, yet not statistically significant. The study findings suggest that initiating early DOACs can lead to a shorter hospital stay without compromising patient safety, as evidenced by consistently low rates of mortality and bleeding [13]. Another retrospective analysis study supports the recommendation of DOAC initiation within less than 48 h among patients with intermediate‐ and high‐risk PE who received thrombolysis. This early initiation of DOACs was associated with a shorter hospital length of stay, as indicated by the study's findings [14].

Whereas DOACs have become clinical guidelines’ first anticoagulation strategy of choice for PE [3, 15], the landmark trials of DOACs versus warfarin VTE/PE treatment have excluded PE patients who required thrombectomy, thrombolysis, or presented with hemodynamic instability [9, 12, 16, 17]. Our meta‐analysis, therefore, aimed to evaluate the effectiveness and safety of DOAC analogs compared to warfarin in patients with intermediate‐ to high‐risk PE. This will potentially approximate the obvious clinical decision “lacuna” that currently subsists in the management of these cohorts of patients.

## 2 Methods

This systematic review and meta‐analysis was conducted adhering to the Preferred Reporting Items for Systematic Reviews and Meta‐Analyses (PRISMA) guidelines. Additionally, the study was registered with PROSPERO under the registration number CRD42024499532.

### 2.1 Study Eligibility Criteria

We included real‐world observational data and randomized controlled trials (RCTs) comparing DOAC analogs versus warfarin in patients diagnosed with intermediate‐ or high‐risk PE. Ethical clearance was unnecessary as this research involved already published secondary data that was accessible in the public domain.

### 2.2 Search Strategy

We conducted a literature search of PubMed, Medline, and EMBASE since their inception till 04/30/2024. No language, date, or article type restrictions were adopted in our search strategy. Example of a database search strategy is: ((((((((((((((((Factor Xa Inhibitors) OR (Dabigatran Etexilate)) OR (Pradaxa)) OR (Rivaroxaban)) OR (Eliquis)) OR (apixaban)) OR (edoxaban tosylate)) OR (direct oral anticoagulants[Title/Abstract])) OR (new oral anticoagulants[Title/Abstract])) OR (NOACs[Title/Abstract])) OR (DOACs[Title/Abstract])) OR (rivaroxaban[Title/Abstract])) OR (dabigatran[Title/Abstract])) OR (apixaban[Title/Abstract])) OR (Edoxaban[Title/Abstract])) AND (((((((warfarin[Title/Abstract]) OR (Acenocoumarol[Title/Abstract])) OR (coumadin[Title/Abstract])) OR (vitamin k antagonist[Title/Abstract])) OR (VK antagonist[Title/Abstract])) OR (warfarin)) OR (apo warfarin[MeSH Terms]))) AND ((((((((((Pulmonary Embolisms[MeSH Terms]) OR (submassive)) OR (massive)) OR (intermediate risk)) OR (high risk)) OR (intermediate‐high risk)) OR (VTE)) OR (submassive[Title/Abstract])) OR (massive[Title/Abstract])) OR (hemodynamically unstable[Title/Abstract])). Additionally, we attempted a manual reference search of retrieved studies.

### 2.3 Inclusion and Exclusion Criteria

Inclusion criteria were as follows: (1) The study had to be either an RCT or observational (prospective or retrospective cohort) study; (2) it should have involved patients diagnosed with intermediate‐risk or high‐risk PE who were prescribed warfarin or DOACs (dabigatran, rivaroxaban, apixaban, or edoxaban); (3) the study needed to report quantitative estimates of hazard ratios (HRs) and 95% confidence intervals (CIs), specifically addressing safety and effectiveness outcomes among these patients. At a minimum, the studies were required to report on VTE recurrence or major bleeding events to be considered for inclusion in the review.

We excluded studies that focused on PE patients but did not include intermediate‐risk or high‐risk PE. Additionally, certain types of publications (e.g., reviews, case reports, case series, letters, and conference abstracts) were excluded due to insufficient data or lack of detailed study information. Pediatric patient cohorts (< 18 years old) and studies that did not meet the inclusion criteria were also excluded.

### 2.4 Screening and Data Extraction

Potential study titles and abstracts were screened initially for eligibility. Eligible articles were retrieved for full‐text review and assessment for inclusion in the review. Two reviewers (M.N.E. and M.A.) performed the search and screening. In case of disagreement between the reviewers, this was resolved by consensus, or a third reviewer (H.A.) adjudicated the disagreement following the protocol. We utilized a predetermined template for retrieving the data. The extracted information encompasses general article information, such as authorship, publication year, study methodology, intervention and control specifics, outcomes, weight, and more.

### 2.5 Outcomes

The primary outcomes of the review are the rate of VTE recurrence and all‐cause mortality. Major and minor bleeding events served as our secondary outcomes (as defined by the primary study authors). We would look at these outcomes at 6 months of follow‐up whenever specified in the study; otherwise, we'll consider the extended observation period where the exposure duration is not specified.

### 2.6 Study Quality and Risk of Bias Assessment

Reviewers assessed the included studies for the risk of bias (ROB) using the Cochrane Risk of Bias Tool for RCTs [18]. Selection bias, performance bias, detection bias, attrition bias, reporting bias, and other biases are the six bias domains covered by the risk of bias tool. The Newcastle Ottawa scale was used to assess the quality of non‐randomized studies [19]. This scale captured eight core elements divided into three broad elements related to the study quality selection, comparability, and exposure. The visualization of the ROBs’ figures was made by Review Manager (RevMan) software V. 5.4 and Risk‐of‐bias Visualization (robvis) software.

### 2.7 Statistical Analysis

The pooled odds ratios (OR) were computed as measures of effect size. The forest plot was generated to summarize the results. Additionally, we conducted a sensitivity analysis to screen for consistency and small‐study effects. The *I^2^* statistic was used to report heterogeneity. An *I* ^2^ > 50% is suggestive of marked heterogeneity in our review. The random‐effects model was used as our meta‐analytical technique. All statistical analyses were performed with STATA software (Stata MP 15) (StataCorp, College Station, TX).

## 3 Results

**FIGURE 1:**
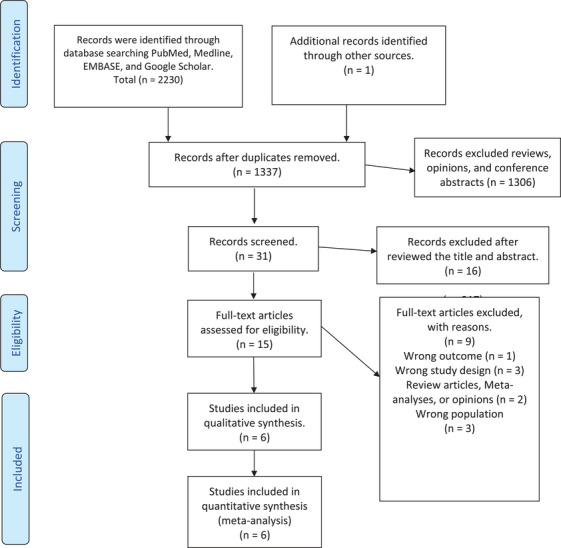
PRISMA flow diagram.

Our exhaustive search strategy retrieved 2231 titles. After screening, we selected 31 titles. Following the abstract review, 15 remaining studies were then subjected to full‐text screenings. Following our predefined inclusion and exclusion criteria, we excluded nine studies for the following reasons: (1) wrong outcome (*n* = 1); (2) wrong study design (*n* = 3); (3) review articles, meta‐analyses, or opinions (*n* = 2); (4) wrong population (*n* = 3). The total number of patients evaluated in these studies is 159,577 patients. The included studies were five observational [14, 20, 21, 22, 23] and one prospective randomized trial [24] meeting our eligibility criteria (Figure 1 shows the PRISMA flow diagram).

Of the six identified studies, consisting of a total of 553 intermediate‐ or high‐risk PE patients who received DOACs or warfarin, one excluded thrombolysis‐treated patients [20], three included only those treated with advanced therapy (systemic thrombolysis [ST] or catheter‐directed thrombolysis [CDT]) [14, 21, 24], and two included both advanced therapy recipients and non‐recipients [22, 23]. Regarding PE risk, two studies by Groetzinger et al. [21] and Santos et al. [20] were confined to intermediate and intermediate‐high‐risk patients, respectively. The remaining four studies included high‐risk patients; however, three did not distinctly report the anticoagulant used and outcomes for each risk category [14, 23, 24]. In the remaining study by Filopei et al. [22], none of the high‐risk patients (*n* = 6) received warfarin. Notably, among intermediate‐risk patients, 8.6% and 10.7% of those receiving DOACs and warfarin, respectively, underwent advanced therapy [22]. The included studies are summarized in (Table 1). Out of the total analyzed cohort, less than 7% of the patients had high‐risk PE (exact number not specified), and 45.7% received advanced therapy [14, 20–24].

**TABLE 1:** Characteristics of PE studies (*n* = 6).

Study author	Country	Design	Anticoagulant, *n* (%)	Mean age, years ± SD, or median, years (IQR)	Male, %	Patients included	Included efficacy outcome	Included safety outcome	Follow‐up‐time
Santos et al. [20]	Portugal	Retrospective cohort	DOAC, 24 (41) Dabigatran, 7 (12) Rivaroxaban, 17 (29) Warfarin, 35 (59)	DOAC, 61 ± 17 Warfarin, 62 ± 19	DOAC, 37 Warfarin, 37	Intermediate‐high risk PE patients (thrombolysis excluded)	Recurrence, mortality	Major bleeding	3 months
Groetzinger et al. [21]	USA	Retrospective cohort	DOAC, 26 (41) Apixaban, 7 (11) Rivaroxaban, 19 (30) Warfarin: 36 (59)	DOAC, 57 (48–64) Warfarin, 61 (44–65)	DOAC, 69 Warfarin, 30	Intermediate‐risk PE patients who required thrombolysis	Recurrence, mortality	Major and minor bleeding	3 months
Filopei et al.^a^ [22]	USA	Retrospective cohort	DOAC, 151 (71) Warfarin, 28 (13) Enoxaparin, 34 (16)	DOAC, 63.7 ± 15.9 Warfarin, 64.4 ± 19.2 Enoxaparin, 56.3 ± 19.2	DOAC, 49 Warfarin, 60.7 Enoxaparin, 35.6	Low, intermediate‐ and high‐risk PE patients (Advanced therapy *n* = 16: DOACs *n* = 13 and Warfarin *n* = 3)	Mortality	Major and minor bleeding	1 months
Lachant et al. [23]	USA	Retrospective and subsequently prospective cohort	DOAC, 70 (71) Apixaban, 37 (38) Rivaroxaban, 33 (34) BMI < 30, 24 BMI 30–39, 27 BMI > 40, 19 Warfarin, 28 (29) BMI < 30, 8 BMI 30‐, 10 BMI > 40, 10	BMI < 30, 69 (98–23) BMI 30–39, 62 (29–85) BMI > 40, 50 (25–75)	BMI < 30, 64 BMI 30–39, 54 BMI > 40, 44	High‐ (*n* = 12) and intermediate‐risk PE patients (*n* = 95) (Advanced therapy *n* = 20)	Recurrence	Major and minor bleeding	2–4 months
Wolfe et al. [14]	USA	Retrospective cohort	DOAC, 53 (52) Apixaban, 24 (23) Rivaroxaban, 29 (28) Warfarin: 39 (38) Enoxaparin, 10 (10)	DOAC, 58.5 ± 14.5 Warfarin, 73 ± 18.3 Enoxaparin, 61 ± 11.7	DOAC, 64.2 Warfarin, 59 Enoxaparin, 30	High‐ and intermediate‐risk PE patients who required thrombolysis SBP median (Q1, Q3) 132 (116, 144 mm Hg), range 62–187 mm Hg	Recurrence, mortality	Major and minor bleeding	3 months
Gostev et al. (RE‐SPIRE) [24]	Russia	Prospective randomized trial	Dabigatran, 32 (51) Warfarin, 31 (49)	Dabigatran, 66 (53–71) Warfarin, 59 (46–67)	Dabigatran, 56.2 Warfarin, 51.6	High‐ and intermediate‐risk PE patients who required thrombolysis	Recurrence, mortality	Major and minor bleeding	6 months

^a^ We reported only intermediate‐risk PE patients' data, as none of the high‐risk patients received warfarin.

### 3.1 Primary Outcomes

#### 3.1.1 VTE Events

**FIGURE 2:**
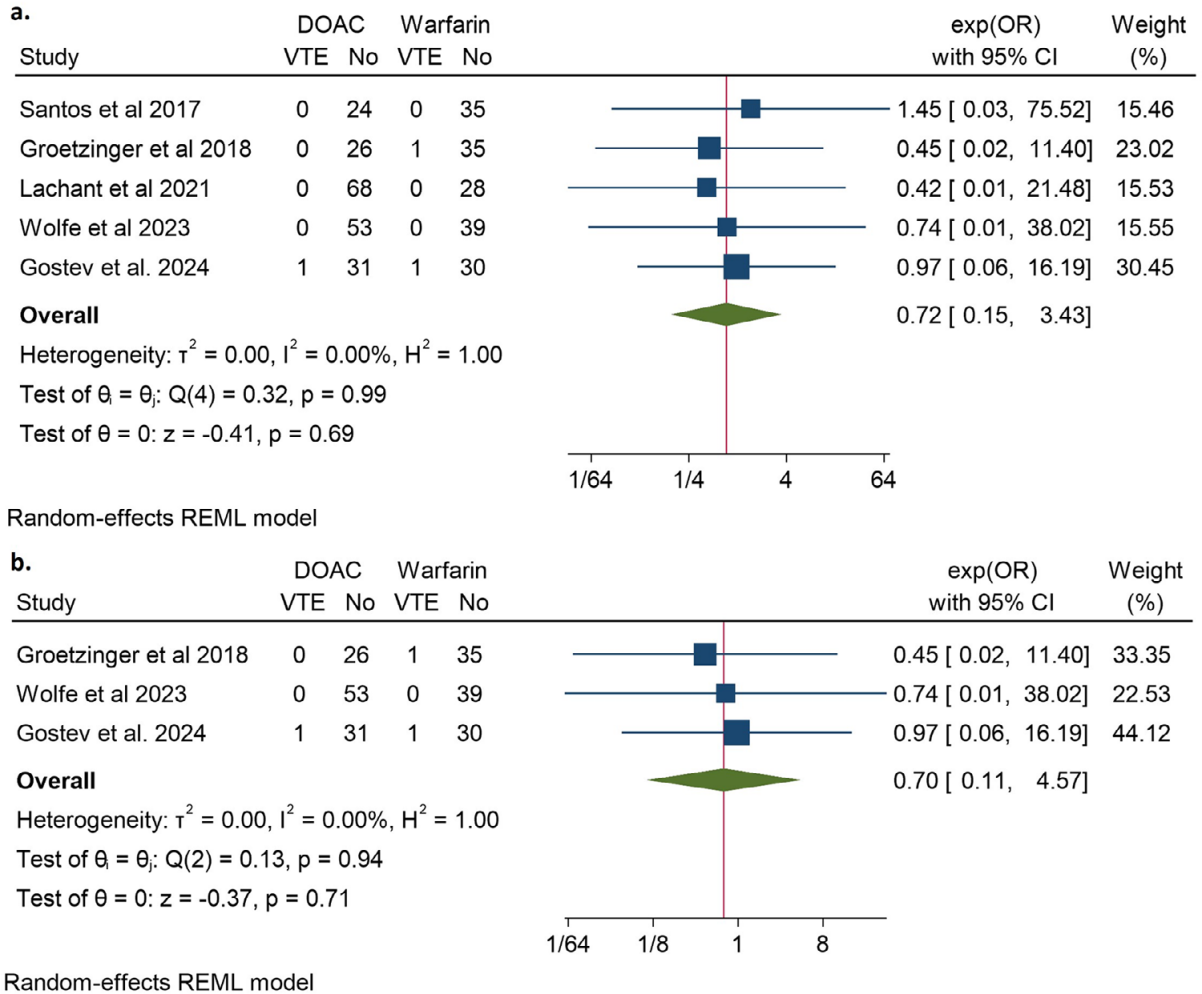
Depicting a forest plot of VTE events in DOAC analogs compared to Warfarin in patients with (a) intermediate‐ to high‐risk PE; (b) intermediate‐ to high‐risk PE post systemic or catheter‐directed thrombolysis only.

Five studies evaluated VTE events in patients with intermediate‐ or high‐risk PE [14, 20, 21, 23, 24]. These studies found no significant difference in VTE events associated with DOAC analogs compared to warfarin (OR 0.72, 95% CI 0.15–3.43, *Q* = 0.32, *I* ^2^ = 0%). The low *I* ^2^ suggested the homogeneity of the results (Figure 2a). Three studies included post‐ST/CDT‐only PE patients [14, 21, 24]. Subgroup analysis of these studies also showed no significant difference in VTE events between DOACs and warfarin (OR 0.7, 95% CI 0.11–4.57, *Q* = 0.13, *I* ^2^ = 0%) (Figure 2b).

#### 3.1.2 Mortality

**FIGURE 3:**
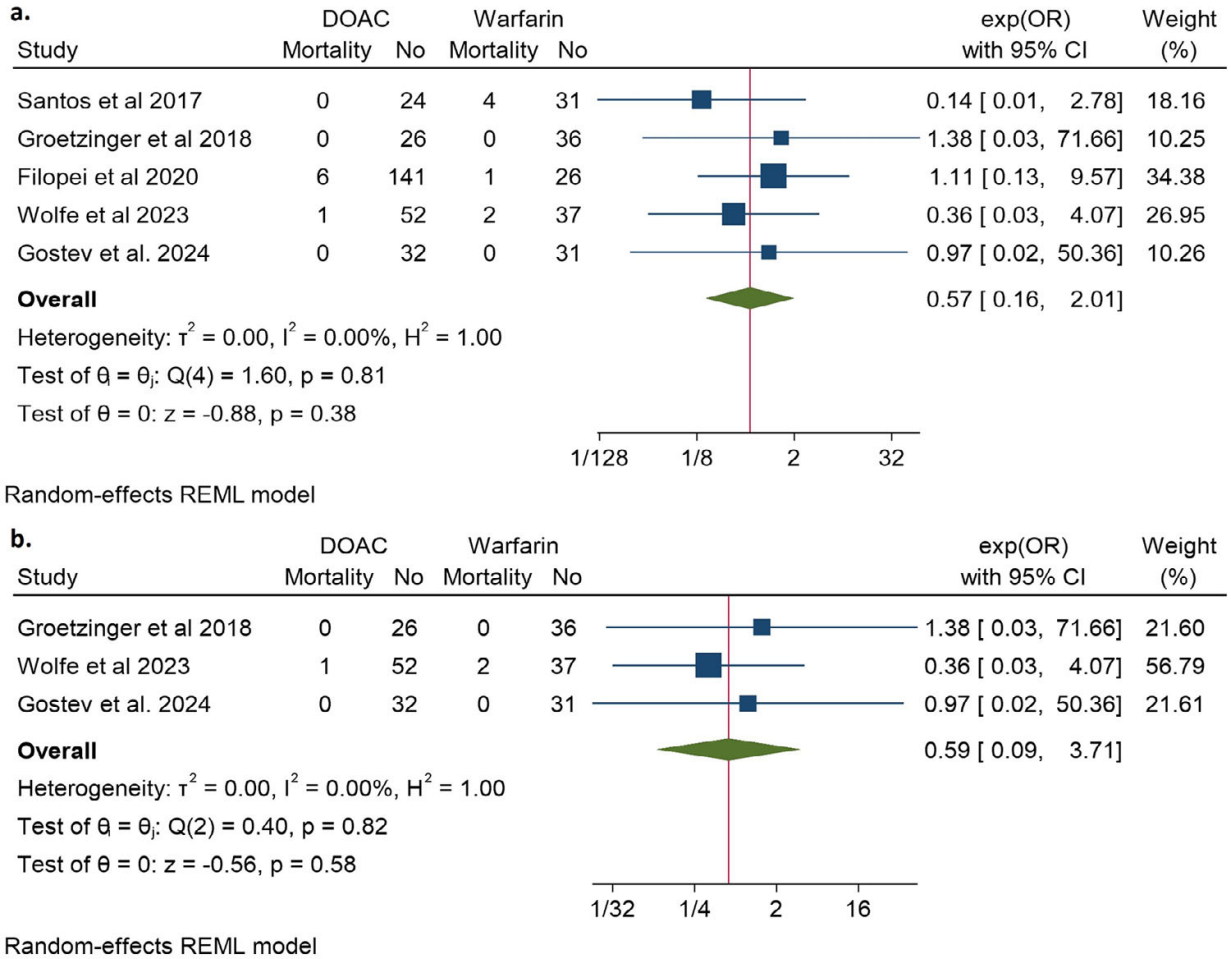
Depicting a forest plot of mortality in DOAC analogs compared to Warfarin in patients with (a) intermediate‐ to high‐risk PE; (b) intermediate‐ to high‐risk PE post systemic or catheter‐directed thrombolysis only.

Five studies evaluated mortality outcomes [14, 20–22, 24]. These studies showed no significant difference in mortality associated with DOACs compared to warfarin. In the pooled analysis of the five studies, patients with intermediate‐ and high‐risk PE who received DOACs had no significant mortality difference versus warfarin (OR 0.57, 95% CI 0.16–2.01, *Q* = 1.6, *I* ^2^ = 0%) (Figure 3a). Subgroup analysis of the post‐ST/CDT‐only studies showed no significant difference in mortality associated with DOACs compared to warfarin (OR 0.59, 95% CI 0.09–3.71, *Q* = 0.4, *I* ^2^ = 0%) (Figure 3b).

### 3.2 Secondary Outcomes

#### 3.2.1 Major Bleeding

**FIGURE 4:**
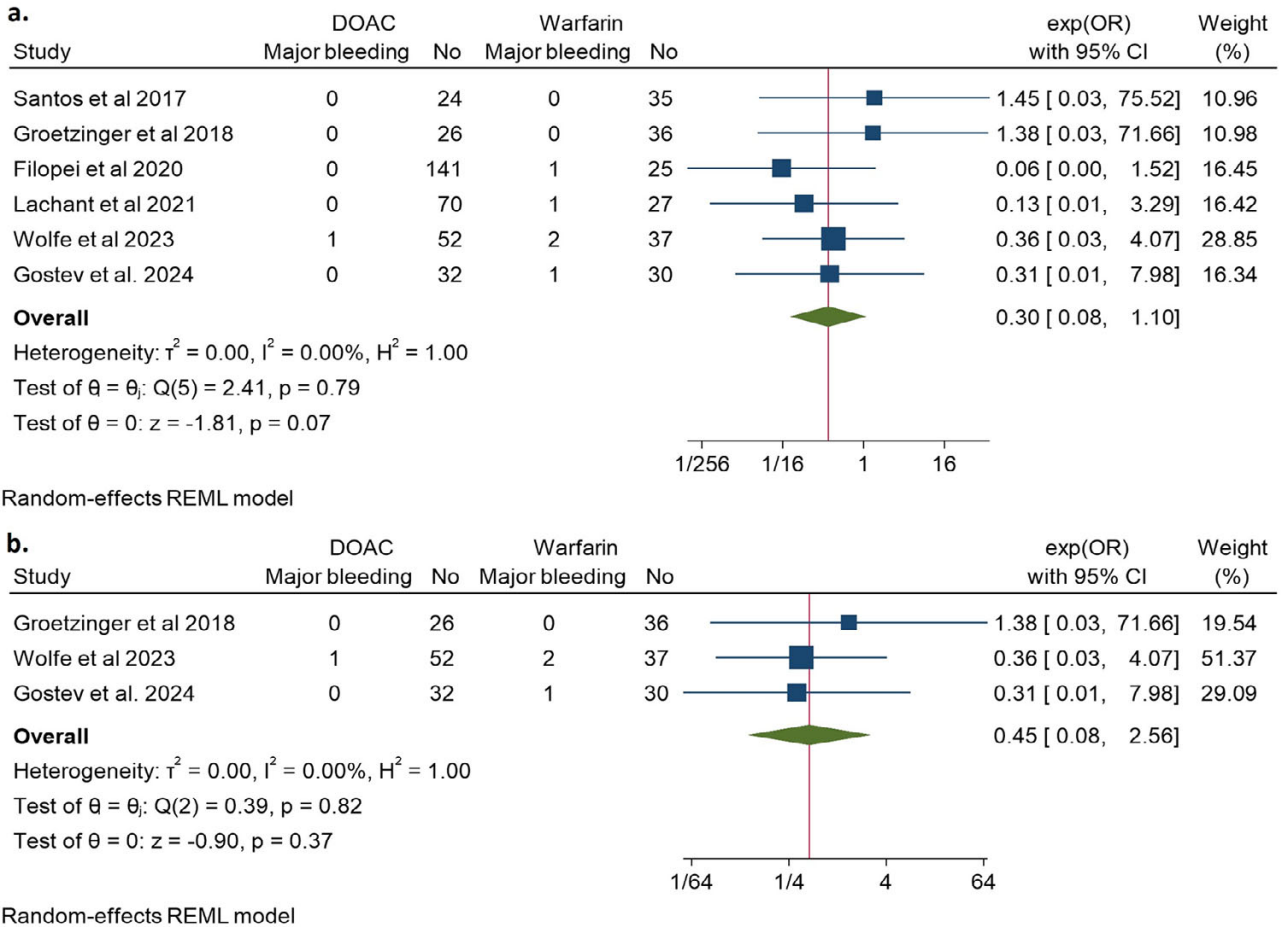
Depicting a forest plot of major bleeding events in DOAC analogs compared to Warfarin in patients with (a) intermediate‐ to high‐risk PE; (b) intermediate‐ to high‐risk PE post systemic or catheter‐directed thrombolysis only.

Six studies evaluated and reported the risk of major bleeding events [14, 20–24]. DOAC analogs had a consistent nonsignificant trend towards an overall reduced risk of major bleeding events by 70% (OR 0.3, 95% CI 0.08–1.1, *Q* = 2.41, *I* ^2^ = 0%) (Figure 4a). Also, for the post‐ST/CDT‐only studies, there was also a nonsignificant trend towards less risk of major bleeding among DOACs versus warfarin (OR 0.45, 95% CI 0.08–2.56, *Q* = 0.39, *I* ^2^ = 0%) (Figure 4b).

#### 3.2.2 Minor Bleeding

**FIGURE 5:**
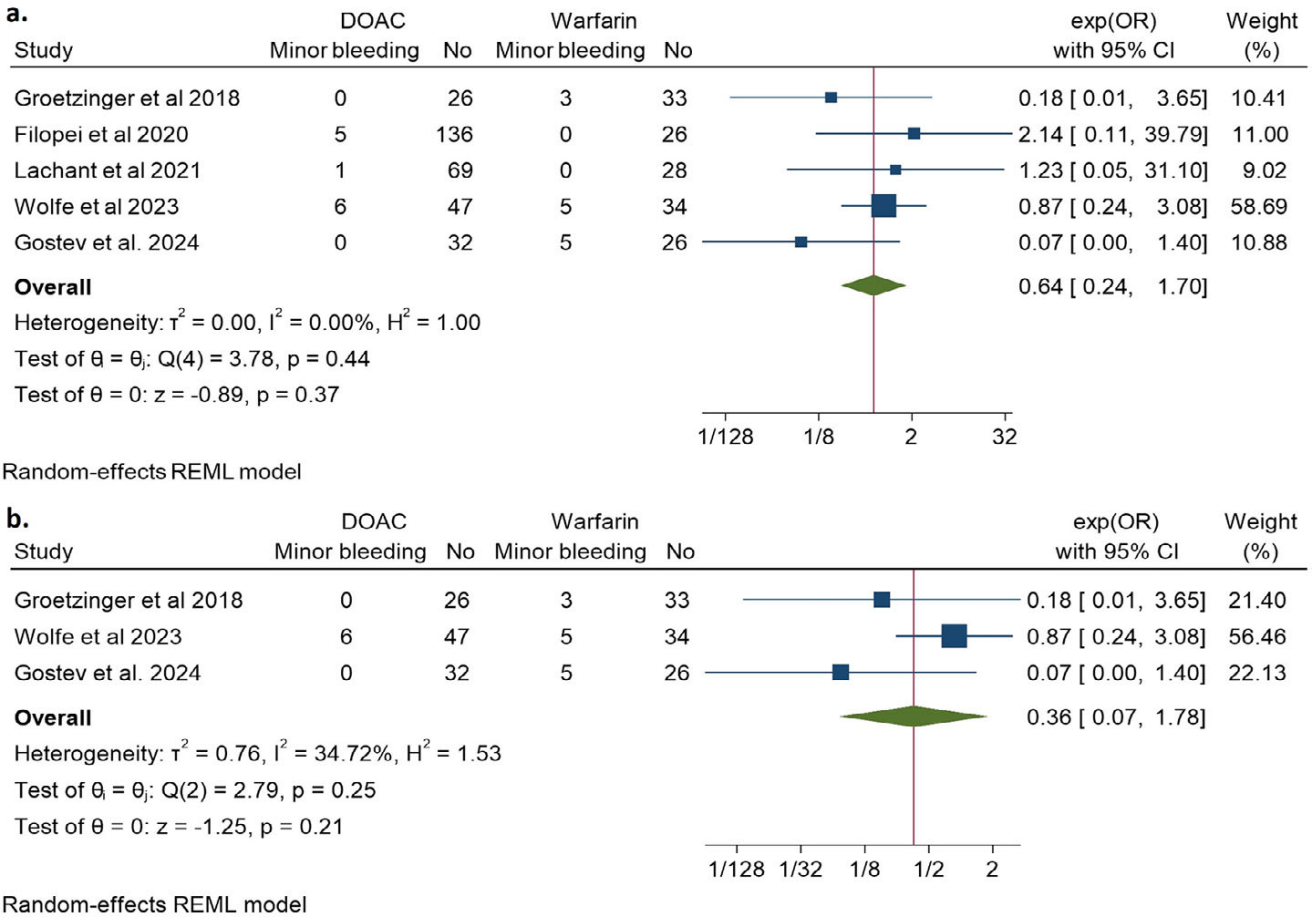
Depicting a forest plot of minor bleeding events in DOAC analogs compared to Warfarin in patients with (a) intermediate‐ to high‐risk PE; (b) intermediate‐ to high‐risk PE post systemic or catheter‐directed thrombolysis only.

Five studies reported minor bleeding events [14, 21–24]. DOAC analogs had a consistent nonsignificant trend towards an overall reduced risk of minor bleeding events by 34% (OR 0.64, 95% CI 0.24–1.7, *Q* = 3.78, *I* ^2^ = 0%) (Figure 5a). The risk was reduced more, yet also nonsignificantly, among the post‐ST/CDT‐only studies by 64% (OR 0.36, 95% CI 0.07–1.78, *Q* = 2.79, *I* ^2^ = 0%) (Figure 5b).

**FIGURE 6:**
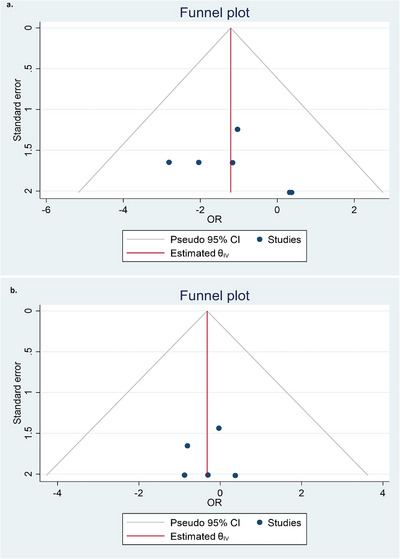
(a) Funnel plot to assess the publication bias for studies assessing VTE events in DOAC analogs versus Warfarin displaying no marked asymmetry; (b) Funnel plot to assess the publication bias for studies assessing major bleeding events in DOAC analogs versus Warfarin showing no marked asymmetry.

The funnel plot revealed no marked asymmetry for VTE and major bleeding events (Figure 6a,b, respectively).

### 3.3 Risk of Bias Assessment

**FIGURE 7:**
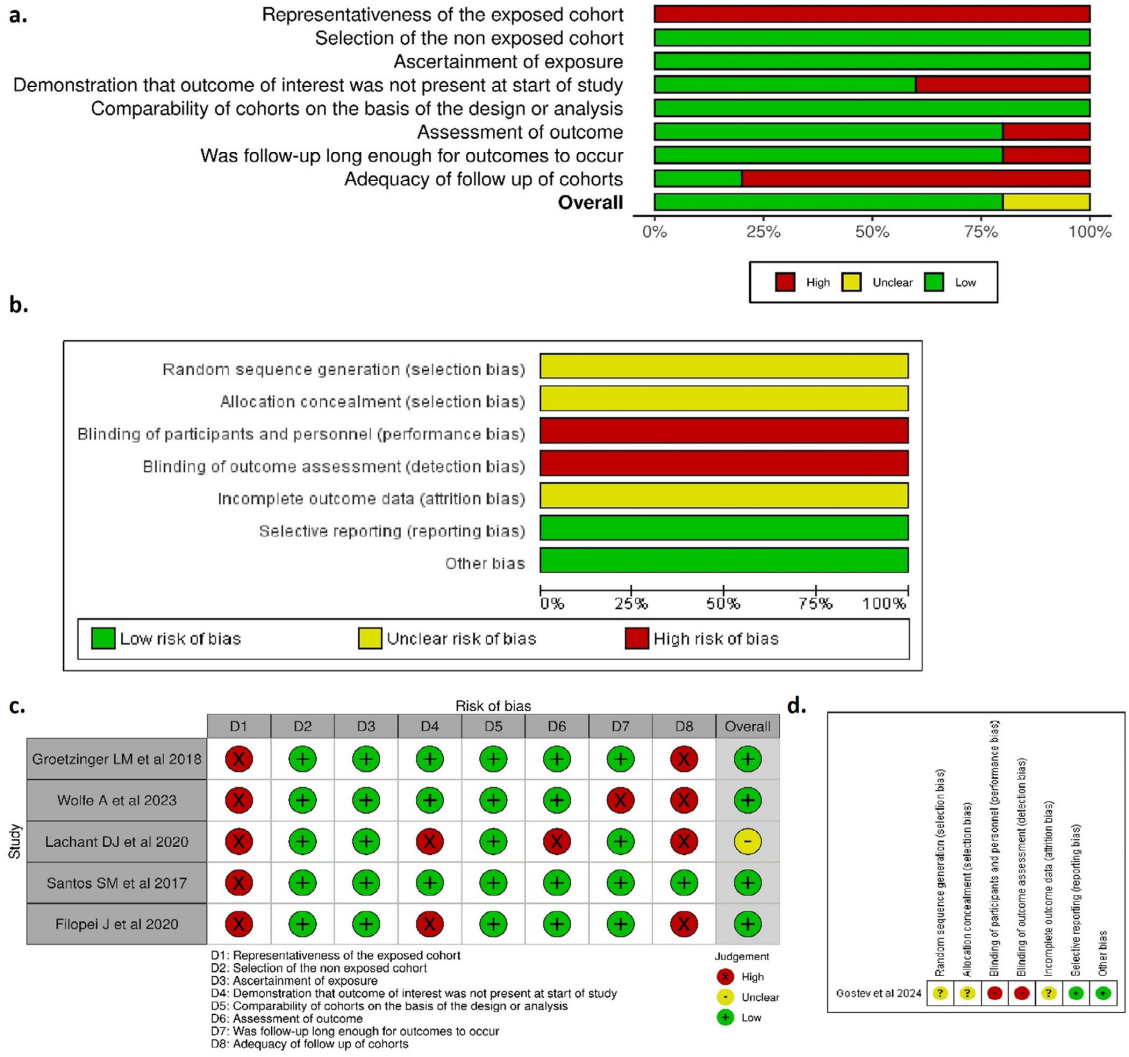
Risk of bias assessment.

The overall quality assessment of cohort studies [14, 20–22] revealed a low risk of bias among all included studies (Figure 7a,c), except Lachant et al. [23] showed an unclear risk of bias. The “Representativeness of the exposed cohort” and “Adequacy of follow‐up of cohorts” items were the leading causes of the high risk of bias. While the only included clinical trial by Gostev et al. [24] showed a high risk of bias (Figure 7b,d).

## 4 Discussion

To our knowledge, this pooled synthesis represents the first comprehensive meta‐analytical review of the differential effect of DOACs compared to warfarin in patients with intermediate‐ or high‐risk PE. This meta‐analysis addresses a critical gap in our understanding of DOAC therapeutics, as previous clinical trials excluded these high‐risk populations, leaving clinicians uncertain about optimal anticoagulation strategies. By synthesizing data from six studies, this work provides valuable insights into the treatment landscape for this vulnerable patient cohort. The inclusion of patients who underwent advanced therapies, such as ST or catheter‐directed interventions, further broadens the applicability of these findings. Despite inherent limitations in the available data, this study highlights the potential of DOACs as a safe and effective alternative to warfarin in these challenging scenarios, encouraging clinicians to consider DOACs for patients with intermediate‐ to high‐risk PE. Our study found no significant differences in VTE recurrence or mortality between DOACs and warfarin for intermediate‐ and high‐risk PE. Subgroup analysis further clarified these outcomes. These findings suggest that DOACs can be a viable alternative, even in patients requiring stabilization through advanced therapies. While high‐risk PE patients were underrepresented in the included studies, the available data indicate similar outcomes between DOACs and warfarin. This suggests that DOACs may be cautiously considered for anticoagulation in stabilized high‐risk PE patients, though further high‐quality research is necessary. Notably, DOACs were associated with trends toward reduced minor bleeding, underscoring their potential benefit in reducing complications in this subgroup.

Among our six analyzed studies, the advanced therapy receivers have been sparingly included by Filopei et al. and Lachant et al. [22, 23], completely excluded by Santos et al. (yet only selected intermediate‐high risk PE) [20], and exclusively included by the other three studies [14, 21, 24]. Groetzinger et al.’s retrospective evaluation of data of 62 patients diagnosed with intermediate‐risk PE who initially had CDT and subsequently stabilized on either DOAC‐based (apixaban or rivaroxaban) versus warfarin‐based anticoagulation strategy conducted over 90 days reported decreased length of hospitalization in the former compared to the latter [21]. Most notably, there were no significant differences in the included efficacy (VTE recurrence) and safety (major bleeding) outcomes between the two groups. The obvious caveat to Groetzinger et al.’s report is the potential interaction effect, given that DOAC‐based therapy was commenced after CDT. It remains, therefore, uncertain what proportion of reported DOAC efficacy and safety outcome estimates from this study were attributable to initial CDT. Understanding the latter is important as a plurality of patients with submassive PE (in real‐world settings) do not require initial CDT before transitioning to oral (DOACs inclusive) or parenteral anticoagulants. The same concern applies to the other two recent post‐thrombolysis‐only studies, by Wolfe et al. and Gostev et al. [14, 24], which both evaluated outcomes of DOACs versus warfarin among high‐ or intermediate‐risk patients post ST or CDT and showed no statistically significant difference in risk of recurrence, mortality, and major bleeding. Additionally, there was a statistically significant more minor bleeding among the warfarin group in the latter study [24]. Our subgroup analysis of those three studies of advanced therapy receivers revealed no difference in efficacy and safety outcomes between DOACs and warfarin. That can shed light on this vulnerable, clinical trials‐excluded cohort.

Additionally, we found a trend towards decreased risks of minor bleeding events amongst patient cohorts stabilized on a strategy of DOAC‐based anticoagulation. High‐risk (massive) PE carries a 90‐day baseline mortality risk of about 52%–58.3% [25], with a significant proportion of this offset by the use of conventional ST. Despite up to a threefold reduction (cf. 15.1% vs. 58.3%) in 90‐day mortality risk, pervading uncertainty regarding therapeutic options spanning both efficacy and safety outcomes in patients with submassive PE has the potential to negate this comparatively low‐risk estimate. The limitation of pharmacotherapy‐based reperfusion strategy (in patients who satisfy indications for it) has largely come down to unacceptably high bleeding risks associated with it, even in select patient groups, an observation generally common with decision‐making around pharmacological anticoagulation. With VKAs and LMWH‐based anticoagulation strategies, bleeding risks have been understandably lower compared to those attributable to thrombolytic therapy. However, until our current review, estimates for both bleeding risks and as efficacy outcomes regarding DOAC‐based strategies in these niche cohorts of patients are not as clearly defined. Despite the improved clinical outcomes of patients with VTE since the advent of the DOAC‐based anticoagulation strategy, their use is still fraught with an annualized major bleeding risk of about 3% [26]. Previous reviews have examined the efficacy and safety of thrombolytic therapy in general in patients with submassive PE. Cao et al. [27], in a systematic review and meta‐analysis of seven studies comprised of 594 patients, showed no disproportionately increased risk of both bleeding risks in patients with submassive PE; this is despite the previously reported high risk of both minor and major hemorrhage in general with the thrombolytic approach.

### 4.1 Strengths and Limitations

Our meta‐analysis supports the consideration of DOACs as a comparable, if not preferable, alternative to warfarin for a select patient cohort with PE, including those at intermediate‐ to high‐risk and patients who require advanced therapy. Whereas the latter category was significantly represented in our pooled analysis, the high‐risk group was only minimally represented and not distinctly reported. The similar efficacy in preventing VTE events and mortality, coupled with a trend towards reduced major and minor bleeding events, highlights the potential benefits of DOACs in clinical practice. Additionally, our meta‐analytical construct sets out the first attempt at a comprehensive examination of secondary published data of DOAC efficacy and safety in these cohorts of patients. This, thus, has the potential to stimulate further attempts at resolving the few remaining clinical uncertainties surrounding anticoagulation strategy in this niche category of patients. However, the observed wide CIs across our outcomes, as well as the limited number of available published studies for review, call for cautious interpretation and underscore the necessity for further large‐scale, high‐quality RCTs to replicate and validate these findings. This synthesis is limited by the numerically small number of published studies explicitly examining the efficacy and safety of DOACs in these “mid‐vulnerable” patient cohorts. This, therefore, calls for caution in the wider interpretability of our review outcomes.

## 5 Conclusion

In patients with intermediate‐ to high‐risk PE stabilized on a DOAC‐based anticoagulation strategy, efficacy and safety outcomes were comparable to those on warfarin, with a lower proportion of both major and minor bleeding events amongst patients on the DOAC‐based strategy. These outcomes were consistent amongst advanced therapy receivers.

## Author Contributions

M.N.E., M.A., M.S., and H.A. agreed on the review idea. M.N.E. and M.A. performed the initial search, screening, and data extraction. M.N.E., M.S., and M.A. extracted data. M.N.E. and M.S. constructed the tables. M.N.E., M.S., and M.D. analyzed the data. M.N.E. produced the figures. M.N.E., M.S., H.A., and M.D. wrote the initial manuscript. The manuscript was then critically revised by M.S. and M.D. All the authors approved the final version of the manuscript for publication.

## Disclosure

The authors have nothing to report.

## Ethics Statement

The authors have nothing to report.

## Consent

The authors have nothing to report.

## Conflicts of Interest

The authors declare no conflicts of interest.

## Data Availability

All the data generated or analyzed during this study are included in the publication article.
